# Knowledge, Attitudes, and Perceptions of African Parents in New Zealand Towards the Human Papillomavirus Vaccine

**DOI:** 10.7759/cureus.76046

**Published:** 2024-12-19

**Authors:** Uje Akoro, George Stuart

**Affiliations:** 1 Medical School, Oceania University of Medicine, Apia, WSM; 2 Research, Oceania University of Medicine, Apia, WSM

**Keywords:** attitude and perceptions, cervical cancer, hpv vaccine, human papillomavirus (hpv), knowledge

## Abstract

Introduction

Human papillomavirus (HPV) is a well-documented cause of cervical cancer, leading to significant mortality that may be decreased through screening and the administration of HPV vaccination. Our understanding of New Zealand immigrants' knowledge, attitudes, and perceptions of the HPV vaccine is limited. Preliminary research shows that immigrants have low knowledge and negative perceptions of the HPV vaccine and that cervical cancer and HPV awareness was low among African parents. This study examines the knowledge, attitudes, and perceptions of African parents in New Zealand towards the HPV vaccine.

Method

A cross-sectional study of 100 African parents was conducted in New Zealand on a randomly selected sample from different African community groups. A self-report questionnaire was used for data collection.

Results

Of the 100 parents who completed the survey, 51 (51%) were women and 49 (49%) were men. Only 45 (45%) of the parents had a high level of knowledge of the HPV vaccine, while 44 (44%) had a low level of knowledge and 11 (11%) had only moderate knowledge. Most of the parents 75 (75%) had a positive attitude toward the HPV vaccine and 71 (71%) had a positive perception of the utilization of the vaccine. A positive relationship was identified between the level of knowledge and attitude (gamma = 0.66, p-value = 0.02) and between the level of knowledge and perception (gamma = 0.73, p-value < 0.001). Parental attitudes towards HPV vaccination uptake were largely influenced by (i) their level of education 88 (88%), (ii) their concerns about the vaccine’s adverse effects 85 (85%), (iii) inadequate knowledge and information about the vaccine 92 (92%), and (iv) public health concerns 89 (89%).

Conclusion

The level of knowledge of the HPV vaccine among 44 (44%) participants in this study was low, though most parents had positive attitudes and perceptions toward the HPV vaccine and were willing to allow their children to be vaccinated against HPV infection. Inadequate knowledge and concerns about the vaccine's adverse effects were some of the main factors influencing the parents' negative attitudes toward the uptake of the HPV vaccine and more education is needed among African parents to improve their knowledge of the HPV vaccine and to address their concerns about the adverse effects of the vaccine. Improved knowledge and education of the HPV vaccine may promote higher vaccine uptake among African parents.

## Introduction

Human papillomavirus (HPV) describes a group of sexually transmitted viruses that can infect both men and women. HPV causes about 99.7% of cervical cancer cases along with penile, anal, and head and neck cancers [1]. Cervical cancer ranks as the fourth most prevalent cancer among women globally and is the third leading cause of mortality, with 570,000 cases and 311,000 deaths recorded in 2018 [2]. Globally, it is a major health concern, especially in sub-Saharan Africa [3]. In New Zealand, around 174 cases and 81 deaths linked to cervical cancer occur annually [4].

The HPV vaccination is safe and well tolerated [5,6] and is the most effective way to prevent HPV infection. The immunity acquired from vaccination contributes not just to a reduction in HPV infections, but also to associated malignancies [7], and the prevention of persistent infections that could develop into invasive cancer [7], as well as protecting against low-risk types of HPV that cause genital warts.

The HPV vaccine was endorsed by the World Health Organisation (WHO) as the main approach to prevent cervical cancer and is most beneficial when administered before first sexual contact. Australia, the United Kingdom, America, and Canada were among the first countries to incorporate the HPV vaccine into their national immunization programs and while the vaccination program in all countries targets young adolescent girls, some countries also target young men in their programs [8]. Most countries administer their vaccines through school-based programs, primary care providers, or health centres [9].

New Zealand introduced HPV vaccination in September 2008 to decrease infection with high-risk types of HPV and its associated cancers. The program offered free vaccination (marketed as Gardasil by Merck) for girls born in 1990 and 1991 and in 2009 it was extended to women born from 1992 onwards. A further extension in January 2017 included men and women under 26 years of age.

The vaccination program aims to achieve herd immunity at a rate that reduces the transmission of HPV infection. Vaccine uptake in New Zealand is currently 68%, and modelling studies suggest that it needs to reach 80% to achieve herd immunity [10]. The lowest uptake rate is amongst Europeans/others (65%), with Māori (68%), Asian (76%), and Pacific people (78%) achieving higher rates [10].

Knowledge, perception, and attitudes towards HPV infection and the HPV vaccine are key factors in increasing vaccine uptake and a better understanding of these factors can help develop targeted campaigns. However, there have been only limited studies of vaccine uptake amongst immigrants, ethnic minorities, and refugees since it became licensed in 2006 [2]. Of the limited studies of the African immigrant population, the majority of findings have come from the United States of America, where studies have shown low knowledge and negative perception of HPV vaccines amongst the immigrant community [2]. HPV and cervical cancer awareness was also generally low amongst African immigrant parents [11], suggesting that these may be linked.

Thus far, no studies have investigated the knowledge, attitudes, and perceptions of immigrant African parents in New Zealand towards the HPV vaccine, yet reporting this data is crucial to maintaining the health and well-being of this group of immigrants. According to official New Zealand government statistics, South Africa alone produces the sixth largest group of international migrants [12]. This study aims to address the research gap by surveying African parents on their knowledge, attitudes, and perceptions towards the HPV vaccine. In line with the US studies, we anticipate that African immigrants living in New Zealand will have low levels of knowledge about and show negative attitudes and perceptions about HPV vaccines. Moreover, we suggest that there will be negative relationships between knowledge of HPV and measures of attitude and perception towards HPV vaccines. Such findings will be important in assisting health authorities in guiding future initiatives to enhance HPV vaccine coverage in New Zealand.

## Materials and methods

Study design

A cross-sectional study of 100 pseudo-randomly selected African parents living in New Zealand. The survey was given to leaders of different groups (e.g. churches and national associations) within the African community who distributed the survey to their members. Respondents returned completed questionnaires to their group leaders who in turn passed them to the researcher.

The participants were informed of the study's aims to determine what African parents living in New Zealand know and feel about the HPV vaccination. Participants were provided with an informed consent form to sign before completing the questionnaire.

Data collection

A self-report questionnaire was used to collect data on socio-demographics, knowledge, attitudes and perceptions about the HPV vaccine amongst African parents and factors affecting attitudes towards the uptake of the HPV vaccine (Appendix). Questions were adopted from validated questionnaires [3,13] and fell into three broad categories. Each section of the questionnaire consisted of five questions: Knowledge of HPV and the vaccine (true/false); attitude towards the vaccine (4-point Likert scale from strongly disagree to strongly agree); perceptions about the purpose of the vaccine (4-point Likert scale from strongly disagree to strongly agree).

Analysis

Data were analysed using non-parametric inferential statistics.

Knowledge

Participants scored 1 for each correct answer and 0 for each incorrect answer. Individual’s knowledge scores were then stratified into high (4 or 5 out of 5), moderate (3 out of 5), and low (1 or 2 out of 5) [14].

Attitude

For each question, participants scored from 1 (negative vaccine attitude) to 4 (positive vaccine attitude). The response value for each question was added to assign an overall value to each participant. Scores exceeding 12/20 were considered to represent a positive attitude, while scores of less than 12/20 were considered to indicate a negative attitude towards the HPV vaccine [14].

Perception

For each question, participants scored from 1 (negative views about the purpose of the HPV vaccine) to 4 (positive views about the purpose of the HPV vaccine). The response value for each question was added to assign an overall value to each participant. Participant scores exceeding 12/20 were considered to represent a positive perception of the utility of the HPV vaccine, while participant scores of less than 12/20 were considered to indicate a negative utility of the HPV vaccine [14].

## Results

Characteristics of the study population

The characteristics of the participants included in the study are summarised in Table 1. The table provides an overview of the demographics and relevant information of the participants. Out of 100 participants, there was a roughly even split between female participants 51 (51%) of the parents and male participants 49 (49%). Most respondents 46 (46%) were aged between 41 and 60 and most 89 (89%) were married or living with a partner. The majority 86 (86%) had been educated up to tertiary standard (Table 1).

**Table 1:** Socio-demographic characteristics of parents in New Zealand

Characteristics	Percentage N (%)
Gender	
Male	49 (49)
Female	51 (51)
Age (years)	
20-40	37 (37)
41-60	46 (46)
61-80	13 (13)
No response	4 (4)
Marital status	
Married or living with a partner	89 (89)
Single	3 (3)
Separated	7 (7)
No response	1 (1)
Educational qualifications	
Primary	2 (2)
Secondary	9 (9)
Tertiary	86 (86)
No response	3 (3)

Knowledge of the HPV vaccine

A relatively large portion of the sample had only low-level knowledge of the HPV vaccine 44 (44%), whereas a similar proportion had a high level of knowledge 45 (45%). This left just 11 (11%) of the sample who we classified as having a moderate level of knowledge. The question most often answered correctly indicated an understanding that the HPV vaccine protects against HPV (67 (67%) of the sample). More than half of the sample were aware that the HPV vaccine cannot be given at any age (53 (53%) of the sample). However, more than half of the sample 56 (56%) did not know that the HPV vaccine requires at least two doses or that the HPV vaccine can be given to both men and women 51 (51%) (Table 2).

**Table 2:** Knowledge of the HPV vaccine HPV: Human papillomavirus

Questions		Correct answer N (%)	Incorrect answer N (%)	Answer “don’t know" N (%)	Missing N
1	The human papillomavirus vaccines protect against infections caused by human papillomaviruses (TRUE)	67 (67)	6 (6)	27 (27)	0
2	The human papillomavirus vaccine requires at least two doses (TRUE)	43 (43)	13 (13)	43 (43)	1
3	The human papillomavirus vaccine offers protection against most cervical cancers (TRUE)	52 (52)	7 (7)	41 (41)	0
4	The human papillomavirus vaccine can be given at any age (FALSE)	53 (53)	8 (8)	38 (38)	1
5	The human papillomavirus vaccine can be given to men and women (TRUE)	49 (49)	18 (18)	33 (33)	0

Attitudes towards the HPV vaccine

The majority of the sample 75 (75%) held a positive attitude towards the HPV vaccine, and this positive bias was consistent across all questions. When specifically asked about the effectiveness of HPV vaccination in preventing HPV infection, 78 (78%) either strongly agreed or agreed (Table 3).

**Table 3:** Attitudes towards the HPV vaccine HPV: Human papillomavirus

Statements		Responses N (%)				
		Strongly agree	Agree	Strongly disagree	Disagree	Blank
1	I have some reservations about the human papillomavirus vaccine	12 (12)	25 (25)	33 (33)	25 (25)	5
2	It is not necessary to be vaccinated against the human papillomavirus	13 (13)	19 (19)	33 (33)	28 (28)	7
3	I feel that the human papillomavirus vaccine is effective in preventing human papillomavirus infection	27 (27)	51 (51)	8 (8)	7 (7)	7
4	I am willing to allow my children to take the human papillomavirus vaccine	22 (22)	43 (43)	12 (12)	18 (18)	5
5	Taking the human papillomavirus vaccine should be encouraged	29 (29)	41 (41)	15 (15)	10 (10)	5

Perceptions towards the HPV vaccine

As with attitudes, the majority of the sample 71 (71%) had a positive perception towards the use and purpose of the HPV vaccine. However, despite most participants either agreeing or strongly agreeing 73 (73%) about the seriousness of their children contracting HPV-related cancer in later life, they were split over the issue of whether their children would be at risk of contracting HPV-related cancers later in life. In fact, half of the participants 50 (50%) either disagreed or strongly disagreed with a statement of this possibility. As to the question of whether pharmaceutical companies were pushing the HPV vaccine to make money, 64 (64%) either disagreed or strongly disagreed (Table 4).

**Table 4:** Perceptions towards the HPV vaccine HPV: Human papillomavirus

Statements		Responses N (%)				
		Strongly Agree	Agree	Disagree	Strongly Disagree	Blank
1	I feel that giving my children the human papillomavirus vaccine would be like performing an experiment on them	13 (13)	20 (20)	47 (47)	17 (17)	3
2	I feel that the human papillomavirus vaccine has many benefits	14 (14)	63 (63)	12 (12)	8 (8)	3
3	I feel that without the human papillomavirus vaccine, my children would be at risk of getting human papillomavirus-related cancers later in life	10 (10)	36 (36)	32 (32)	18 (18)	4
4	I feel that it would be serious if my children contracted a human papillomavirus-related cancer later in life	20 (20)	53 (53)	12 (12)	12 (12)	3
5	The human papillomavirus vaccine is being pushed to make money for pharmaceutical companies	10 (10)	18 (18)	51 (51)	13 (13)	8

Factors believed to influence the uptake of the HPV vaccine

When asked to speculate about the reasons for parental uptake of the HPV vaccine, most participants believed that important factors were the level of education (88 (88%) agreed or strongly agreed) and protection of public health (89 (89%) agreed or strongly agreed). Factors that were believed to make uptake less likely included concerns about adverse effects of the vaccine (85 (85%) agreed or strongly agreed), inadequate knowledge or information about the vaccine (92 (92%) agreed or strongly agreed), and distance to a vaccination centre (56 (56%) agreed or strongly agreed, 41 (41%) disagreed or strongly disagreed, and three blank responses) (Table 5).

**Table 5:** Factors influencing the attitude of parents towards the uptake of the HPV vaccine HPV: Human papillomavirus

Statements		Responses N (%)				
		Strongly Agree	Agree	Disagree	Strongly Disagree	Blank
1	The level of education can affect the use of the human papillomavirus vaccine	40 (40)	48 (48)	6 (6)	5 (5)	1
2	Concerns about adverse effects can affect the adoption of the human papillomavirus vaccine	24 (24)	61 (61)	9 (9)	5 (5)	1
3	Distance to a vaccination centre is a barrier to the use of the human papillomavirus vaccine	8 (8)	48 (48)	26 (26)	15 (15)	3
4	Inadequate knowledge or information about the human papillomavirus vaccine can affect the adoption	56 (56)	36 (36)	3 (3)	3 (3)	2
5	Vaccines are a good way to protect public health	51 (51)	38 (38)	4 (4)	6 (6)	1

Relationship between knowledge, attitudes and perception

The ordinal data were analysed using Goodman-Kruskal gamma tests [15] to evaluate the strength of the relationship between the level of knowledge and both attitude towards the vaccine and accuracy of perceived purpose of the HPV vaccine. There was a positive trend between the level of knowledge and attitude (from negative to positive), gamma = 0.66, p = 0.002, indicating that greater knowledge was associated with more positive attitudes (Table 6). Similarly, there was a significant positive relationship between the level of knowledge and perception of the purpose of the vaccine (from negative to positive), gamma = 0.73, p < 0.001 (Table 6). The calculated gamma values for attitude and perception levels were close to 1 indicating a strong positive trend.

**Table 6:** Knowledge, attitude and perception levels towards HPV vaccination Knowledge levels were stratified into three categories: low, moderate and high knowledge. Attitude and perception levels were stratified into two categories: negative and positive. ± Test of ordinal trend based on gamma statistic.

	Attitude levels ±		Perception levels ±	
	Gamma	p-value	Gamma	p-value
Knowledge levels	0.66	0.002	0.73	<0.001

## Discussion

In common with other studies of immigrant communities elsewhere in the world [11,16], we found that just under half of our New Zealand sample had low-level knowledge of the HPV vaccine and this indicates that there is more work to be done, at least in educating this particular cohort. However, there may be lessons to be learnt from the Nigerian experience where Nigerian parents were reported to have had a high level of knowledge about the HPV vaccine [3].

It was reassuring to find that most of the parents in this survey demonstrated a positive attitude toward HPV vaccines and this finding aligns with several other studies [17,18]. Many of the parents in our study felt that the HPV vaccine effectively prevents HPV infection and were willing to allow their children to be vaccinated against HPV; however, at least one study reported negative parental attitudes towards the HPV vaccine [19], which should caution against complacency.

In common with other studies of immigrant communities showing that most mothers were willing to have their daughters vaccinated against HPV if it would protect or improve their health [20], the majority of parents in our study had a positive perception of the purpose and use of the HPV vaccine. This is in contrast to a negative perception observed amongst an African community in England, where the HPV vaccine was seen as incompatible with culture and religion which influenced risk perception toward the vaccine and played an important role in vaccination decision-making [11].

In our study, parents' attitudes towards the uptake of the HPV vaccination were largely influenced by their level of education, concerns about the vaccine's adverse effects, lack of knowledge about the vaccine, and the need for protection of public health. Safety concerns, inadequate knowledge about the vaccine, level of education, and distance to a vaccination centre have all been found to impact vaccination uptake in a similar study looking at Nigerian parents in Nigeria [3].

Overall, like other studies [14], the results presented here demonstrate a positive relationship between knowledge, attitude and perception which emphasises the importance of education amongst immigrant communities, even in nations such as New Zealand, with robust vaccination and screening initiatives. Thus, health professionals play a crucial role in addressing this knowledge gap [1].

Limitations

The study's limitations include the challenge of applying the results to the wider African population of New Zealand due to the small sample size utilised. Additionally, the cross-sectional design of the study, which gathers data at a single point in time, contributes to this limitation and future studies are necessary both to increase the sample and understand the variability over time.

## Conclusions

The study showed that less than half of the participants had a low level of knowledge about the HPV vaccine, and most parents had positive attitudes and perceptions towards the HPV vaccine. Factors affecting the attitude of the participants towards the uptake of the HPV vaccine were the level of education, concerns about adverse effects, inadequate knowledge or information about the HPV vaccine and protection of public health. Inadequate knowledge was the major factor affecting uptake. Education about the HPV vaccine should be encouraged among African parents. This may help to increase their knowledge of the vaccine and address concerns about potential side effects.

## Appendices

Knowledge, attitudes and perceptions of African parents in New Zealand towards the human papillomavirus vaccine.

Please answer the following questions to the best of your knowledge. 

Part 1: Socio-Demographic Information

Please tick the boxes that apply to you

Who is completing the questionnaire?

☐Mother

☐Father

☐Female guardian

☐Male Guardian

What is your age

☐20-40

☐41-60

☐61-80

☐Other (please specify)

What is your marital status

☐Married or living with partner

☐Single

☐Divorced, separated or widowed

What is your highest level of educational qualification

☐Primary

☐Secondary

☐Tertiary

☐None

☐Other (please specify)

Part 2: Knowledge of Human Papillomavirus Vaccine

The following statements will assess your knowledge of the human papillomavirus vaccine. Please read and tick one of the options: True, False or Don’t Know.  

**Table 7:** Knowledge of human papillomavirus vaccine

	True	False	Don’t Know
The human papillomavirus vaccine protects against infections caused by human papillomaviruses.			
The human papillomavirus vaccine requires at least two doses.			
The human papillomavirus vaccine offers protection against most cervical cancers.			
The human papillomavirus vaccine can be given at any age.			
The human papillomavirus vaccine can be given to males and females.			

Part 3: Attitude towards the Human Papillomavirus Vaccine

The following statements will examine your attitude towards the human papillomavirus vaccine. Please read and tick one of the options: Strongly Agree, Agree, Disagree or Strongly Disagree. 

I wish to know your opinion. Please note that I am not testing your knowledge. If you do not have an answer, that’s alright, simply select the answer that most reflects your opinion.

**Table 8:** Attitude towards the human papillomavirus vaccine

	Strongly Agree	Agree	Disagree	Strongly Disagree
I have some reservations about the human papillomavirus vaccine.				
It is not necessary to be vaccinated against the human papillomavirus.				
I feel that the human papillomavirus vaccine is effective in preventing human papillomavirus infection.				
I am willing to allow my children take the human papillomavirus vaccine.				
Taking the human papillomavirus vaccine should be encouraged.				

Part 4: Perceptions of the Human Papillomavirus Vaccine

The following statements will examine your perceptions of the human papillomavirus vaccine. Please read and tick one of the options: Strongly Agree, Agree, Disagree or Strongly Disagree.

I wish to know your opinion. Please note that I am not testing your knowledge. If you do not have an answer, that’s alright, simply select the answer that most reflects your opinion.

**Table 9:** Perceptions of the human papillomavirus vaccine

	Strongly Agree	Agree	Disagree	Strongly Disagree
I feel that giving my children the Human Papillomavirus Vaccine would be like performing an experiment on them.				
I feel that the Human Papillomavirus Vaccine has many benefits.				
I feel that without the Human Papillomavirus Vaccine, my children would be at risk of getting Human Papillomavirus related cancers later in life.				
I feel that it would be serious if my children contracted a Human Papillomavirus related cancer later in life.				
The Human Papillomavirus Vaccine is being pushed to make money for pharmaceutical companies.				

Part 5: Factors Influencing Attitude of Parents Towards the Uptake of the Human Papillomavirus Vaccine

The following statements will examine likely factors affecting the uptake of the human papillomavirus vaccine. Please read and tick as appropriate by using any of these options: Strongly Agree, Agree, Disagree or Strongly Disagree

**Table 10:** Factors influencing attitude of parents towards the uptake of the human papillomavirus vaccine

	Strongly Agree	Agree	Disagree	Strongly Disagree
The level of Education can affect the use of the human papillomavirus vaccine.				
Concern about adverse effects can affect the adoption of the human papillomavirus vaccine.				
Distance to a vaccination centre is a barrier to the use of the human papillomavirus Vaccine.				
Inadequate knowledge or information about the human papillomavirus vaccine can affect the adoption.				
Vaccines are a good way to protect public health.				

Thank you for participating in this survey.

## References

[1] Sherman SM, Bartholomew K, Denison HJ, Patel H, Moss EL, Douwes J, Bromhead C (2018). Knowledge, attitudes and awareness of the human papillomavirus among health professionals in New Zealand. PLoS One.

[2] Netfa F, Tashani M, Booy R, King C, Rashid H, Skinner SR (2020). Knowledge, attitudes and perceptions of immigrant parents towards human papillomavirus (HPV) vaccination: a systematic review. Trop Med Infect Dis.

[3] Ohareri B, Adefolaju AO, Onyeneho CA (2020). Knowledge, attitudes and perceptions of Nigerian parents towards human papilloma virus (HPV) vaccines. Eur J Midwifery.

[4] Bruni L, Albero G, Serrano B (2024). Human Papillomavirus and Related Diseases Report in New Zealand. October.

[5] Garland SM, Cornall AM, Brotherton JM, Wark JD, Malloy MJ, Tabrizi SN (2018). Final analysis of a study assessing genital human papillomavirus genoprevalence in young Australian women, following eight years of a national vaccination program. Vaccine.

[6] Brotherton JM, Bloem PN (2018). Population-based HPV vaccination programmes are safe and effective: 2017 update and the impetus for achieving better global coverage. Best Pract Res Clin Obstet Gynaecol.

[7] Cervantes JL, Doan AH (2018). Discrepancies in the evaluation of the safety of the human papillomavirus vaccine. Mem Inst Oswaldo Cruz.

[8] (2022). Human papillomavirus vaccines: WHO position paper. Weekly Epidemiological Record No 50.

[9] Gallagher KE, LaMontagne DS, Watson-Jones D (2018). Status of HPV vaccine introduction and barriers to country uptake. Vaccine.

[10] (2024). Human Papillomavirus Vaccination: A RESEARCH REVIEW™ Hesitancy and Uptake. www.researchreview.co.nz. Published online.

[11] Mupandawana ET, Cross R (2016). Attitudes towards human papillomavirus vaccination among African parents in a city in the north of England: a qualitative study. Reprod Health.

[12] New Zealand Government (2024 (2024). International Migration: January 2024. International migration: January.

[13] Sherman SM, Nailer E (2018). Attitudes towards and knowledge about Human Papillomavirus (HPV) and the HPV vaccination in parents of teenage boys in the UK. PLoS One.

[14] Hourani L, Zaatar M, Hoballah J (2024). Overview of knowledge, attitudes and barriers associated with HPV vaccination in Beirut, Lebanon. Glob Public Health.

[15] Goodman LA, Kruskal WH (1954). Measures of association for cross classifications. J Am Stat Assoc.

[16] Grandahl M, Tydén T, Gottvall M, Westerling R, Oscarsson M (2015). Immigrant women's experiences and views on the prevention of cervical cancer: a qualitative study. Health Expect.

[17] Aragones A, Genoff M, Gonzalez C, Shuk E, Gany F (2016). HPV vaccine and Latino immigrant parents: if they offer it, we will get it. J Immigr Minor Health.

[18] Glenn BA, Tsui J, Singhal R (2015). Factors associated with HPV awareness among mothers of low-income ethnic minority adolescent girls in Los Angeles. Vaccine.

[19] Albright K, Barnard J, O'Leary ST (2017). Noninitiation and noncompletion of HPV vaccine among English- and Spanish-speaking parents of adolescent girls: a qualitative study. Acad Pediatr.

[20] Stephens DP, Thomas TL (2013). Cultural values influencing immigrant Haitian mothers' attitudes toward human papillomavirus vaccination for daughters. J Black Psychol.

